# Association between red cell distribution width and its ratio with albumin and diabetic nephropathy/retinopathy: A systematic review and meta-analysis

**DOI:** 10.12669/pjms.41.10.12622

**Published:** 2025-10

**Authors:** Lejuan Ma, Chungen Yan, Shufang Lu, Yali Xing

**Affiliations:** 1 Lejuan Ma School Health Office, Affiliated Hospital of Shaoxing University, Shaoxing, Zhejiang Province 312000, P.R. China; 2 Chungen Yan Department of Gastroenterology, Affiliated Hospital of Shaoxing University, Shaoxing, Zhejiang Province 312000, P.R. China; 3 Shufang Lu Neurological Chronic Disease Rehabilitation Department, Affiliated Hospital of Shaoxing University, Shaoxing, Zhejiang Province 312000, P.R. China; 4 Yali Xing Department of Geriatric Medicine II, Affiliated Hospital of Shaoxing University, Shaoxing, Zhejiang Province 312000, P.R. China

**Keywords:** Biomarkers, Diabetic Retinopathy, Diabetic nephropathy, Diabetes Mellitus

## Abstract

**Objective:**

Red cell distribution width (RDW) and RDW-albumin ratio (RAR) have been used as biomarkers for various illnesses. We reviewed published literature to assess if RDW and RAR can be predictors of predictors of diabetic nephropathy (DN) and diabetic retinopathy (DR).

**Methods:**

As per PRISMA guidelines, observational studies published on the databases of PubMed, Embase, Scopus and Web of Science till 20^th^ December 2024 were included. We determined the pooled odds ratio (OR) for the association between RDW/RAR and DN/DR.

**Results:**

Nine studies on RDW and three studies (four cohorts) on RAR were included. Studies on RDW and RAR included 6347 and 5005 patients respectively. Meta-analysis showed that high RDW was associated with higher odds of DN (OR: 1.70 95% CI: 1.39, 2.07 I^2^=43%) but not DR (OR: 1.18 95% CI: 0.97, 1.44 I^2^=35%). Association between RDW and DN did not change in sensitivity and subgroup analyses. Meta-analysis also found that high RAR was significantly associated with higher odds of DN (OR: 2.74 95% CI: 1.30, 5.74 I^2^=92%). Data from one study found no relationship between RAR and DR.

**Conclusions:**

Higher RDW and RAR are associated with higher odds of DN. However, both the markers may not be associated with DR. In view of limited data, results must be interpreted with caution till further studies are available.

## INTRODUCTION

Diabetes mellitus (DM) is a global health concern. Type-2 DM (T2DM) is the predominant subtype of the disease and is mostly attributable to lifestyle habits with a genetic predisposition.^1^ The disease incurs a significant healthcare expenditure with expenses to the tune of 966 billion USD in 2021 which could rise to 1,054 billion USD by 2045.^2^ It’s not only the disease per se but also the related morbidity that contributes to the high healthcare costs. Uncontrolled sugar levels in DM can have serious consequences affecting several organ systems leading to debilitating complications.^3^ Macrovascular and microvascular complications namely cardiovascular disorders, diabetic nephropathy (DN), diabetic retinopathy (DR) and cardiovascular autonomic neuropathy can cause significant disability, impaired quality of life and increased mortality.^4^

A meta-analysis study from China shows that about 21.8% of T2DM patients suffer from DN and it is a leading cause of end-stage renal disease.^5^ Likewise, the prevalence of DR in diabetics in China is estimated to be 18.5%.^6^ Given the high prevalence of these conditions, there is a need for simple but accurate markers that can predict the risk of DN and DR. Availability of such markers can help identify patients at higher risk of complications, allowing earlier diagnosis and treatment to slow the progression of the disease.^7^

Widely researched and simple biomarker often discussed in literature is the red blood cell distribution width (RDW) which signifies variation in the size of erythrocytes in a blood sample. Its value is expressed in percentage, with higher values indicating greater variability in cell size.^8^ A large number of studies have investigated the potential role of RDW as a prognostic marker in several diseases like cardiac disorders (atrial fibrillation, heart failure, coronary heart disease), pulmonary embolism, sepsis, kidney and liver disease, stroke and malignancies.^9^-^15^

The advantage of the marker is its ease of availability and low cost allowing easy risk stratification of patients even in lower-income countries.^16^ The potential role of RDW as a biomarker has been attributed to its ability to reflect systemic inflammation.^9^-^15^ To further improve its prognostic ability, it has been recently combined with albumin, another marker of inflammation and malnutrition.^17^ The RDW-albumin ratio (RAR) has also been used to predict the prognosis of several illnesses.^17^,^18^ However, their value in predicting complications of T2DM is still underexplored. We therefore examined if RDW and RAR can be used to predict complications of T2DM, namely DN and DR.

## METHODOLOGY

The review followed the PRISMA guidelines.^19^ It was registered on PROSPERO (CRD42024628088). All studies relevant to the review topic were explored on the databases of PubMed, Embase, Scopus and Web of Science. The literature search was from the inception of the above-mentioned databases to 20^th^ December 2024 and was conducted by two reviewers independently (LM, CY). In addition to these databases, the reference lists of eligible articles, relevant reviews and Google Scholar was scanned for potential studies. Only English language publications were considered. A mix of keywords and MeSH words were used for narrowing our search. Details are shown in Supplementary Table-I.

**Supplementary Table-I:** Search protocol.

Query	Search Details
(((red cell distribution width) OR (RDW)) AND (diabetes)) AND (microvascular complications)	("erythrocyte indices"[MeSH Terms] OR ("erythrocyte"[All Fields] AND "indices"[All Fields]) OR "erythrocyte indices"[All Fields] OR ("red"[All Fields] AND "cell"[All Fields] AND "distribution"[All Fields] AND "width"[All Fields]) OR "red cell distribution width"[All Fields] OR "RDW"[All Fields]) AND ("diabete"[All Fields] OR "diabetes mellitus"[MeSH Terms] OR ("diabetes"[All Fields] AND "mellitus"[All Fields]) OR "diabetes mellitus"[All Fields] OR "diabetes"[All Fields] OR "diabetes insipidus"[MeSH Terms] OR ("diabetes"[All Fields] AND "insipidus"[All Fields]) OR "diabetes insipidus"[All Fields] OR "diabetic"[All Fields] OR "diabetics"[All Fields] OR "diabets"[All Fields]) AND (("microvascular"[All Fields] OR "microvascularity"[All Fields] OR "microvascularization"[All Fields] OR "microvascularized"[All Fields]) AND ("complicances"[All Fields] OR "complicate"[All Fields] OR "complicated"[All Fields] OR "complicates"[All Fields] OR "complicating"[All Fields] OR "complication"[All Fields] OR "complication s"[All Fields] OR "complications"[MeSH Subheading] OR "complications"[All Fields]))
(((red cell distribution width) OR (RDW)) AND (diabetes)) AND (((((nephropathy) OR (kidney disease)) OR (proteinuria)) OR (albuminuria)) OR (urinary albumin creatinine ratio))	("erythrocyte indices"[MeSH Terms] OR ("erythrocyte"[All Fields] AND "indices"[All Fields]) OR "erythrocyte indices"[All Fields] OR ("red"[All Fields] AND "cell"[All Fields] AND "distribution"[All Fields] AND "width"[All Fields]) OR "red cell distribution width"[All Fields] OR "RDW"[All Fields]) AND ("diabete"[All Fields] OR "diabetes mellitus"[MeSH Terms] OR ("diabetes"[All Fields] AND "mellitus"[All Fields]) OR "diabetes mellitus"[All Fields] OR "diabetes"[All Fields] OR "diabetes insipidus"[MeSH Terms] OR ("diabetes"[All Fields] AND "insipidus"[All Fields]) OR "diabetes insipidus"[All Fields] OR "diabetic"[All Fields] OR "diabetics"[All Fields] OR "diabets"[All Fields]) AND ("kidney diseases"[MeSH Terms] OR ("kidney"[All Fields] AND "diseases"[All Fields]) OR "kidney diseases"[All Fields] OR "nephropathies"[All Fields] OR "nephropathy"[All Fields] OR ("kidney diseases"[MeSH Terms] OR ("kidney"[All Fields] AND "diseases"[All Fields]) OR "kidney diseases"[All Fields] OR ("kidney"[All Fields] AND "disease"[All Fields]) OR "kidney disease"[All Fields]) OR ("proteinuria"[MeSH Terms] OR "proteinuria"[All Fields] OR "proteinurias"[All Fields]) OR ("albuminuria"[MeSH Terms] OR "albuminuria"[All Fields]) OR (("urinary tract"[MeSH Terms] OR ("urinary"[All Fields] AND "tract"[All Fields]) OR "urinary tract"[All Fields] OR "urinary"[All Fields]) AND ("albumin s"[All Fields] OR "albumine"[All Fields] OR "albumines"[All Fields] OR "albumins"[MeSH Terms] OR "albumins"[All Fields] OR "albumin"[All Fields]) AND ("creatinin"[All Fields] OR "creatinine"[MeSH Terms] OR "creatinine"[All Fields] OR "creatinines"[All Fields]) AND ("ratio"[All Fields] OR "ratio s"[All Fields] OR "ratioes"[All Fields] OR "ratios"[All Fields])))
(((red cell distribution width) OR (RDW)) AND (diabetes)) AND (((retinopathy) OR (eye disease)) OR (vision))	("erythrocyte indices"[MeSH Terms] OR ("erythrocyte"[All Fields] AND "indices"[All Fields]) OR "erythrocyte indices"[All Fields] OR ("red"[All Fields] AND "cell"[All Fields] AND "distribution"[All Fields] AND "width"[All Fields]) OR "red cell distribution width"[All Fields] OR "RDW"[All Fields]) AND ("diabete"[All Fields] OR "diabetes mellitus"[MeSH Terms] OR ("diabetes"[All Fields] AND "mellitus"[All Fields]) OR "diabetes mellitus"[All Fields] OR "diabetes"[All Fields] OR "diabetes insipidus"[MeSH Terms] OR ("diabetes"[All Fields] AND "insipidus"[All Fields]) OR "diabetes insipidus"[All Fields] OR "diabetic"[All Fields] OR "diabetics"[All Fields] OR "diabets"[All Fields]) AND ("retinal diseases"[MeSH Terms] OR ("retinal"[All Fields] AND "diseases"[All Fields]) OR "retinal diseases"[All Fields] OR "retinopathies"[All Fields] OR "retinopathy"[All Fields] OR ("eye diseases"[MeSH Terms] OR ("eye"[All Fields] AND "diseases"[All Fields]) OR "eye diseases"[All Fields] OR ("eye"[All Fields] AND "disease"[All Fields]) OR "eye disease"[All Fields]) OR ("vision s"[All Fields] OR "vision, ocular"[MeSH Terms] OR ("vision"[All Fields] AND "ocular"[All Fields]) OR "ocular vision"[All Fields] OR "vision"[All Fields] OR "visions"[All Fields] OR "visioning"[All Fields]))
(((red cell distribution width albumin ratio) OR (RAR)) AND (diabetes)) AND (((retinopathy) OR (eye disease)) OR (vision))	((("erythrocyte indices"[MeSH Terms] OR ("erythrocyte"[All Fields] AND "indices"[All Fields]) OR "erythrocyte indices"[All Fields] OR ("red"[All Fields] AND "cell"[All Fields] AND "distribution"[All Fields] AND "width"[All Fields]) OR "red cell distribution width"[All Fields]) AND ("albumin s"[All Fields] OR "albumine"[All Fields] OR "albumines"[All Fields] OR "albumins"[MeSH Terms] OR "albumins"[All Fields] OR "albumin"[All Fields]) AND ("ratio"[All Fields] OR "ratio s"[All Fields] OR "ratioes"[All Fields] OR "ratios"[All Fields])) OR "RAR"[All Fields]) AND ("diabete"[All Fields] OR "diabetes mellitus"[MeSH Terms] OR ("diabetes"[All Fields] AND "mellitus"[All Fields]) OR "diabetes mellitus"[All Fields] OR "diabetes"[All Fields] OR "diabetes insipidus"[MeSH Terms] OR ("diabetes"[All Fields] AND "insipidus"[All Fields]) OR "diabetes insipidus"[All Fields] OR "diabetic"[All Fields] OR "diabetics"[All Fields] OR "diabets"[All Fields]) AND ("retinal diseases"[MeSH Terms] OR ("retinal"[All Fields] AND "diseases"[All Fields]) OR "retinal diseases"[All Fields] OR "retinopathies"[All Fields] OR "retinopathy"[All Fields] OR ("eye diseases"[MeSH Terms] OR ("eye"[All Fields] AND "diseases"[All Fields]) OR "eye diseases"[All Fields] OR ("eye"[All Fields] AND "disease"[All Fields]) OR "eye disease"[All Fields]) OR ("vision s"[All Fields] OR "vision, ocular"[MeSH Terms] OR ("vision"[All Fields] AND "ocular"[All Fields]) OR "ocular vision"[All Fields] OR "vision"[All Fields] OR "visions"[All Fields] OR "visioning"[All Fields]))
(((red cell distribution width albumin ratio) OR (RAR)) AND (diabetes)) AND (((((nephropathy) OR (kidney disease)) OR (proteinuria)) OR (albuminuria)) OR (urinary albumin creatinine ratio))	((("erythrocyte indices"[MeSH Terms] OR ("erythrocyte"[All Fields] AND "indices"[All Fields]) OR "erythrocyte indices"[All Fields] OR ("red"[All Fields] AND "cell"[All Fields] AND "distribution"[All Fields] AND "width"[All Fields]) OR "red cell distribution width"[All Fields]) AND ("albumin s"[All Fields] OR "albumine"[All Fields] OR "albumines"[All Fields] OR "albumins"[MeSH Terms] OR "albumins"[All Fields] OR "albumin"[All Fields]) AND ("ratio"[All Fields] OR "ratio s"[All Fields] OR "ratioes"[All Fields] OR "ratios"[All Fields])) OR "RAR"[All Fields]) AND ("diabete"[All Fields] OR "diabetes mellitus"[MeSH Terms] OR ("diabetes"[All Fields] AND "mellitus"[All Fields]) OR "diabetes mellitus"[All Fields] OR "diabetes"[All Fields] OR "diabetes insipidus"[MeSH Terms] OR ("diabetes"[All Fields] AND "insipidus"[All Fields]) OR "diabetes insipidus"[All Fields] OR "diabetic"[All Fields] OR "diabetics"[All Fields] OR "diabets"[All Fields]) AND ("kidney diseases"[MeSH Terms] OR ("kidney"[All Fields] AND "diseases"[All Fields]) OR "kidney diseases"[All Fields] OR "nephropathies"[All Fields] OR "nephropathy"[All Fields] OR ("kidney diseases"[MeSH Terms] OR ("kidney"[All Fields] AND "diseases"[All Fields]) OR "kidney diseases"[All Fields] OR ("kidney"[All Fields] AND "disease"[All Fields]) OR "kidney disease"[All Fields]) OR ("proteinuria"[MeSH Terms] OR "proteinuria"[All Fields] OR "proteinurias"[All Fields]) OR ("albuminuria"[MeSH Terms] OR "albuminuria"[All Fields]) OR (("urinary tract"[MeSH Terms] OR ("urinary"[All Fields] AND "tract"[All Fields]) OR "urinary tract"[All Fields] OR "urinary"[All Fields]) AND ("albumin s"[All Fields] OR "albumine"[All Fields] OR "albumines"[All Fields] OR "albumins"[MeSH Terms] OR "albumins"[All Fields] OR "albumin"[All Fields]) AND ("creatinin"[All Fields] OR "creatinine"[MeSH Terms] OR "creatinine"[All Fields] OR "creatinines"[All Fields]) AND ("ratio"[All Fields] OR "ratio s"[All Fields] OR "ratioes"[All Fields] OR "ratios"[All Fields])))

### Inclusion Criteria:

- Two authors selected eligible studies according to the following inclusion criteria:
- All observation study designs were conducted on T2DM.
- Studies reporting the association between RDW or RAR with DN or DR.
- Studies reporting the effect size of the association for a meta-analysis.

### Exclusion criteria:

- Studies not reporting data on DN or DR.
- Studies not reporting outcomes with 95% confidence intervals (CI).
- Studies not specifically on T2DM.
- Studies available only as abstracts and unpublished data.

The two reviewers combined the search results in a single reference manager module and removed duplicate studies. Both reviewers then screened each study by their title and abstract to retain relevant articles and exclude non-relevant studies. Any study found relevant to either reviewer was retrieved and analyzed by reading its complete text. Articles found fulfilling the inclusion criteria by both reviewers were retained for the study. The authors resolved differences through detailed discussion with the third reviewer (SL).

Two reviewers (CY & SL) extracted relevant data for the review in duplicate. Details pertaining to the author’s name, study type, location, demographic details of the sample, hypertension, smoking, diabetes duration, complication assessed (DN or DR), its definition, RDW or RAR cut-off used and effect size data were sourced by the reviewers.

Given the observational nature of studies, we utilized the Newcastle Ottawa Scale (NOS) to judge the quality of studies.^20^ Two reviewers (SL & YX) examined the studies in detail based on questions of the NOS and marked the articles on a score of zero to nine. Studies with scores of >8 were considered high-quality studies. The reviewers discussed and resolved any disagreements.

A quantitative synthesis of data was conducted using “Review Manager” (RevMan, version 5.3). We first segregated studies based on the marker used: RDW or RAR. Data on DN or DR was then pooled to generate an odds ratio (OR) with 95% CI which indicated the combined risk of the complication based on higher values of RDW or RAR. If a study reported data of multiple subgroups, they were combined using the meta-analysis software. Heterogeneity among studies was assessed through Cochran’s Q statistic and the I^2^ index. I^2^ of over 50% and/or P < 0.05 indicated a large degree of heterogeneity.

Nevertheless, due to methodological variations between studies, the inverse variance random-effect model was chosen irrespective of the quantified inter-study heterogeneity. For meta-analyses with ≥4 studies, a sensitivity analysis was conducted to assess the credibility of the results. Studies were removed sequentially from the meta-analysis and the pooled OR was re-calculated. Funnel plots were not plotted given the small number of studies in the meta-analysis. Subgroup analysis was conducted for the meta-analysis of RDW and DN based on location, study design and RDW cut-off.

## RESULTS

**Fig.1:**
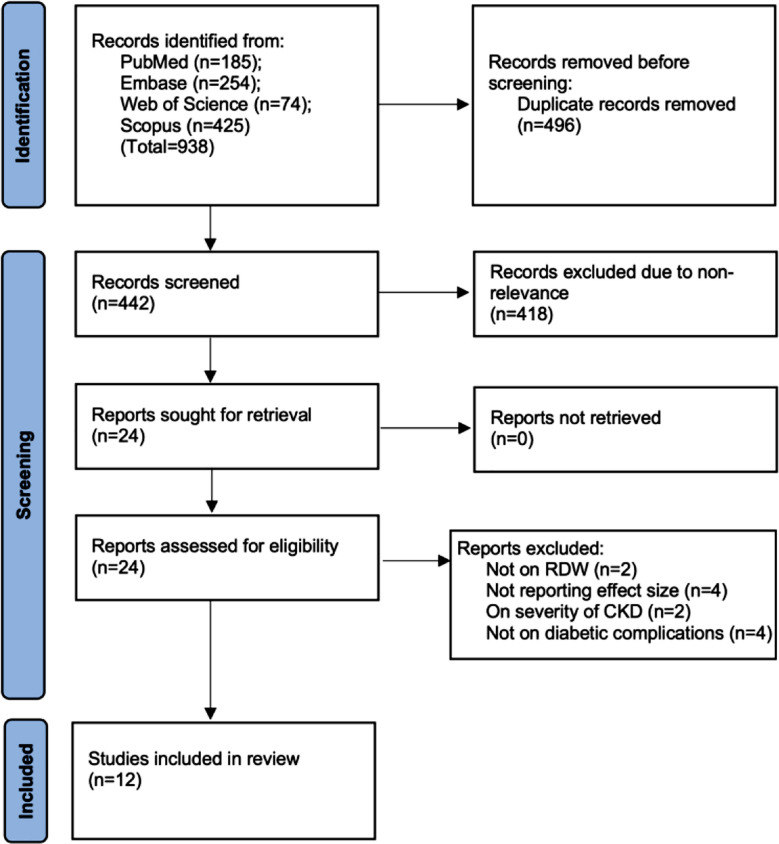
Study flowchart based on PRISMA showing the screening process.

Results of all databases and the screening process are displayed in Fig.1. In the end, 12 studies were included of which nine^21^-^29^ were on RDW and three on RAR.^30^,^31^ High inter-reviewer agreement was noted (kappa=0.95). Characteristics of studies on RDW are presented in Table-I. In terms of study design, most of the studies were cross-sectional, one was a case-control study while three were cohort studies. The nine studies on RDW included a total of 6347 patients. Diabetes duration varied significantly in the included studies. One study assessed both DN and DR. Six other studies examined only DN while two reported data on DR. Diagnosis of DN was based on objective parameters involving urinary protein and creatinine in all studies. DR was also diagnosed either by an ophthalmologist or by objective parameters. The cut-off of RDW varied from 12.2 to 14.6 in the included studies. After assessment of the methodology of studies, the reviewers marked two studies with a score of seven and the remaining with a score of eight.

**Table-I:** Characteristics of studies on RDW.

Study	Country	Type	Sample size	Male (%)	Age (years)	HT (%)	Smoking (%)	Diabetes duration	Complication	Definition	RDW cut-off	NOS score
Xi 2021^21^	China	RC	1095	56.9	NR	41.7	28	>20 years: 3.1%; 10-20 years: 33.9	Nephropathy	UACR> 30mg/g	13	7
Ma 2021^22^	China	CC	298	58	48.5	45.5	1.7	9.9± 7 years	Retinopathy	Diagnosed by ophthalmologist	12.2	8
Roumeliotis 2020^23^	Greece	RC	142	54.2	68	NR	21.1	25.3± 7.8 years	Nephropathy	Based on the National Kidney Foundation Kidney Disease Outcomes Quality Initiative criteria	14.6	8
Baslov 2018^24^	Croatia	PC	247	54.9	56	83	NR	11.2± 1.3 years	Retinopathy	Diagnosed by ophthalmologist	NR	7
Al-Rubeaan 2018^25^	Saudi Arabia	CS	640	44.5	56.6	74.5	NR	18.2± 5.9 years	Nephropathy	UACR> 30mg/g	NR	8
Xiong 2017^26^	China	CS	809	54.3	60	NR	30.7	10± 1 years	Nephropathy	Proteinuria more than 30 mg/L	12.4	8
Zhang 2015^27^	China	CS	410	47.3	46.8	23.9	11.4	NR	Nephropathy	UACR> 30mg/g	12.8	8
Magri 2014^28^	Malta	CS	209	39.2	64.8	NR	9	18.3± 9.5	Nephropathy	Microalbuminuria (UACR ≥ 2.5 mg/mmol in men or 3.5 mg/ mmol in women) or macroalbuminuria (i.e. an UACR ≥30 mg/mmol)	NR	8
Malandrino 2012^29^	USA	CS	2497	44.5	60	77.4	20.6	9.9± 8 years	Retinopathy Nephropathy	Modified Airlie House Classification scheme; UACR ≥3.4 mg/mmol	12.6	8

UACR, urinary albumin to creatinine ratio; RDW, red cell distribution width; HT, hypertension; NOS, Newcastle Ottawa scale; RC, retrospective cohort; PC, prospective cohort; CC, case-control; CS, cross-sectional; NR, not reported.

Details of studies on RAR are shown in Table-II. Two cohorts were cross-sectional while the remaining two were retrospective. A total of 5005 patients were included in the studies. Three reported data only on DN while one was on both DN and DR. DN was diagnosed based on urinary albumin to creatinine ratio in all studies. The cut-off of RAR ranged from 3.2 to 3.3 in the studies. All studies were high quality with a NOS score of eight.

**Table-II:** Characteristics of studies on RAR.

Study	Country	Type	Sample size	Male (%)	Age (years)	HT (%)	Smoking (%)	Diabetes duration	Complication	Definition	RAR cut-off	NOS score
Yu 2024^30^	China	CS	427	57.8	60	52.5	NR	9 years	Retinopathy Nephropathy	Diagnosed by ophthalmologist UACR ≥30 mg/g and/or eGFR<60 mL/min/1.73 m² for >3 months	3.28	8
Chen 2024^31^	USA	CS	2839	51.9	60.8	NR	NR	3± 8.6 years	Nephropathy	UACR ≥30 mg/g and/or eGFR<60mL/min/1.73 m²	3.3	8
	China	RC	1412	53	63	NR	NR	8.6± 2.1 years	Nephropathy	UACR ≥30 mg/g and/or eGFR<60mL/min/1.73 m²	3.2	8
Tutan 2023^32^	Turkey	RC	327	45.2	61	NR	NR	NR	Nephropathy	Spot UACR ≥ 0.3	3.3	8

eGFR, estimated glomerular filtration rate; UACR, urinary albumin to creatinine ratio; RAR, red cell distribution width-albumin ratio; HT, hypertension; NOS, Newcastle Ottawa scale; RC, retrospective cohort; CS, cross-sectional; NR, not reported.

### Association between RDW and DN/DR:

**Fig.2:**
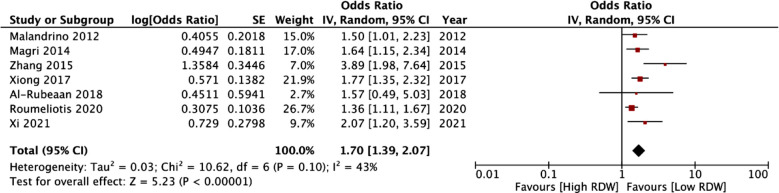
Forest plot depicting the association between RDW and DN.

**Fig.3:**
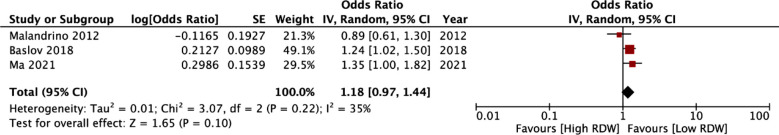
Forest plot depicting the association between RDW and DR.

Meta-analysis of seven studies reporting the association between RDW and DN showed that high RDW was associated with higher odds of DN (OR: 1.70 95% CI: 1.39, 2.07 I^2^=43%) (Fig.2). A sensitivity analysis was conducted to explore the effect of each study on the pooled association. (Supplementary Table-II). Sequential exclusion of studies did not alter the significance of the results. The heterogeneity was reduced to 0% on the exclusion of the study of Zhang et al.^27^ Dividing the studies based on location, we noted statistically significant results for both Western and Asian studies (Supplementary Table-III). Subgroup analysis based on study design also failed to change the significance of the outcomes. Likewise, a significant association was noted for studies using an RDW cut-off of ≥13% as well as <13%. Three studies reported the association between RDW and DR. Pooled analysis of data showed that high RDW values were not associated with a higher risk of DR (OR: 1.18 95% CI: 0.97, 1.44 I^2^=35%) (Fig.3).

**Supplementary Table-II:** Sensitivity analysis for the association between RDW and nephropathy.

Removed article	Resultant odds ratio and 95% confidence intervals	I^2^
Malandrino 2012	1.76 [1.39, 2.24]	52
Magri 2014	1.74 [1.36, 2.23]	53
Zhang 2015	1.55 [1.35, 1.77]	0
Xiong 2017	1.71 [1.33, 2.21]	50
Al-Rubeaan 2018	1.72 [1.39, 2.12]	53
Roumeliotis 2020	1.82 [1.48, 2.24]	22
Xi 2021	1.67 [1.35, 2.07]	49

**Supplementary Table-III:** Subgroup analysis of the association between RDW and nephropathy.

Variable	Groups	Studies	OR [95% CI]	I^2^
Location	Asian	4	2.12 [1.50, 2.99]	36
	Western	3	1.44 [1.22, 1.69]	0
Study type	Cross-sectional	5	1.81 [1.41, 2.31]	35
	Retrospective cohort	2	1.55 [1.06, 2.27]	50
Cut-off	<13%	3	1.98 [1.31, 2.99]	66
	≥13%	3	1.44 [1.19, 1.74]	1

OR, odds ratio; CI, confidence intervals

### Association between RAR and DN/DR:

**Fig.4:**
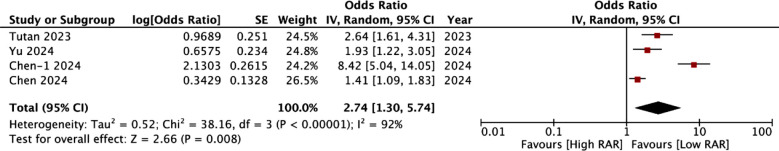
Forest plot depicting the association between RAR and DN.

Data was available from four cohorts for the association between RAR and DN. Meta-analysis found that high RAR was significantly associated with odds of DN (OR: 2.74 95% CI: 1.30, 5.74). High inter-study heterogeneity was seen for this analysis (I^2^=92%) (Fig.4). Sensitivity analysis was conducted in the software itself and no change in the significance of the results was noted on removal of any cohort. Only one study of Yu et al^30^ reported the association between RAR and DR. The authors noted that high RAR quartile did not significantly increase the likelihood of developing DR (OR: 1.18 95% CI 0.63-2.21, p = 0.59).

## DISCUSSION

Prediction of diabetic complications has been a subject of intense research in the past decade.^32^,^33^ This is primarily because complications like DN and DR are major causes of end-stage renal disease and blindness respectively causing significant disability and reduction in quality of life.^4^ DN is also associated with a concomitant increase in the risk of cardiovascular disease and mortality which may be prevented by early identification of the disease using appropriate biomarkers.^34^ On one hand, there are sophisticated markers like cystatin C, neutrophil gelatinase-associated lipocalin, plasma KIM-1, fibroblast growth factors 21 and 23 and pigment epithelium-derived factor^35^ and on the other hand there are a plethora of prediction models, nomograms, model equations, machine learning models, risk score charts and neural networks which have been investigated for predicting diabetic complications.^36^,^37^ While some of these markers and scores have high accuracy in predicting the risk of DN and DR, they may not be suitable for routine use, especially in resource-limited settings. Therefore, there is a need for simpler biomarkers that are easily available for routine monitoring of diabetic patients.

RDW is one such marker that has been linked with the development of DM. Gang et al^38^ in a retrospective cohort study of 2,688 individuals from the general population found that those with the highest quartile of RDW had an 80% increased risk of developing DM even after adjustment of multiple confounders. Another retrospective study on 2703 Chinese adults also found that every unit increase in RDW was associated with a 16% increase in the risk of DM. The authors suggested that RDW can be an inexpensive, noninvasive and convenient indicator that may be incorporated into DM prediction models.^39^ Overall, DM patients have been shown to have higher RDW values as compared to the general population and the highest values are often noted in uncontrolled diabetics.^40^ This association has also been extrapolated to predict the risk of diabetic complications. High RDW has been linked with the development of diabetic ketoacidosis, cardiovascular mortality as well as all-cause mortality in DM patients.^41^,^42^ RDW has also been combined with albumin to generate RAR which is postulated to further improve the prediction of outcomes.^17^,^18^ Research shows that RAR could be a suitable indicator for peripheral artery disease and lower extremity ulcers in diabetic patients.^43^,^44^

However, the value of RDW and RAR in predicting the risk of the most morbid complications namely DN and DR is still unclear. Since most of the research in the literature has focused on DN and DR, this present review aims to summarize data from published studies to present the best possible evidence for clinical practice. We were able to retrieve nine studies on RDW and three studies with four cohorts on RAR. Meta-analysis showed that higher values of RDW were associated with increased risk of DN but not DR. Likewise, very limited data showed a significant association between RAR and DN but a single study^30^ found no association between RAR and DR. The lack of association between RDW and RAR with DR is indeed perplexing and may be explained by two reasons.

Firstly, the number of studies reporting data on DR was not high. With just three studies on RDW and one on RAR, the statistical power may not have been high enough to demonstrate significant associations. Secondly, the difference in pathophysiological mechanisms between DN and DR may have contributed to varying results. Both micro and macrovascular mechanisms are involved in the pathogenesis of DN as opposed to mostly microvascular disease in DR.^29^ One may argue that inflammation has also been implicated in the pathogenesis of DR^6^ and RDW is a marker of inflammation.^9^-^15^ However, research shows a weak relationship between DR and systemic inflammatory markers like C-reactive protein and therefore it is plausible that the inflammatory process related to DR may be more localized and not related to systemic inflammation.^29^,^45^,^46^ Since RDW is reflective of systemic inflammation which affects the macrovasculature,^9^-^15^ it may partially explain the lack of predictive ability of DR.

The common link between RDW, albumin and DM are inflammation. DM and metabolic syndrome are diseases associated with chronic inflammation. Higher baseline systemic inflammation status has been noted in individuals who develop DM later in life. Furthermore, high concentrations of inflammatory markers have been seen in uncontrolled DM and those developing complications.^47^ Systemic inflammation is linked with ineffective maturation of erythrocytes, reduced iron metabolism and decreased cell survival which cause an increase in RDW.^48^,^49^ A positive association between inflammatory markers like interleukin-6, C-reactive protein and erythrocyte sedimentation rate has been seen with increased RDW.^50^

Additionally, the inflammatory mediator, tumor necrosis factor leads to hypoferremia which causes erythrophagocytosis. Cytokines also lead to deformities in RBC membranes suppressing erythrocyte maturation. This leads to the production of larger reticulocytes causing an increase in RDW.^9^-^15^ Particularly in DM, increased glycosylation of cell surface proteins, reduced plasma membrane fluidity and decreased erythrocyte deformability make the erythrocytes vulnerable to damage further increasing RDW.^30^ While RDW as a singular factor reflects the systemic inflammatory status, the addition of albumin improves the prognostic ability of the marker.

This was noted in our meta-analysis wherein RAR had a higher OR as compared to RDW for the prediction of DN. Albumin is considered a marker for systemic inflammation and malnutrition.^43^,^44^ Serum albumin levels alone have been associated with incident diabetes and diabetic microvascular complications.^51^ Furthermore, malnourished patients have a higher risk of both DN and DR and albumin may be an efficient marker of the baseline nutritional status.^52^,^53^ Given the small number of studies on RAR, further research is needed to establish its superiority over RDW particularly in predicting diabetic complications.

### Strength of the study:

It is the first systematic review and meta-analysis in the literature that examines the role of RDW and RAR in predicting diabetic complications. We conducted a detailed literature search and combined peer-reviewed data to provide the best available evidence. Wherever possible, sensitivity and subgroup analyses were conducted to better interpret the results.

### Limitations:

Despite the pathophysiological links between RDW, RAR and diabetic complications, the results of the current review must be interpreted with caution. Firstly, the number of studies available for the meta-analysis was not high. The study designs were also mostly cross-sectional which does not establish a causal relationship between RDW and complications. Segregation of data based on study design was only possible for the meta-analysis of RDW and DN which demonstrated significant results for all study types. Nevertheless, only two cohort studies were available. Secondly, unlike RAR, the cut-off of RDW showed wide variation in the included studies. While subgroup analysis demonstrated significant results for studies using a cut-off of ≥13% and <13%, the present review was unable to determine the best cut-off to predict DN or DR. Thirdly, despite most studies reporting adjusted outcomes, there is always a possibility of unmeasured confounders affecting the study results. Lastly, the included studies were from a few specific countries and do not represent global data. This prohibits the generalization of evidence.

## CONCLUSIONS

Higher RDW and RAR are associated with higher odds of DN. However, both the markers may not be associated with DR. Based on the outcomes, we can suggest that RDW and RAR can be used for recognizing patients at higher risk of DN. They may be used as an inexpensive and readily available screening tools for selecting patients for further investigations. Future prospective studies are needed to validate the current results.

### Authors’ contributions:

**LM:** Study design, literature search and manuscript writing.

**LM, CY, SL and YX:** Data collection, data analysis and interpretation. Critical Review.

**LM:** Was involved in the manuscript revision and validation and is responsible for the integrity of the study.

All authors have read and approved the final manuscript.
